# Complete Genome Sequence of *Lactobacillus curvatus* Strain WiKim38 Isolated from Kimchi

**DOI:** 10.1128/genomeA.00273-17

**Published:** 2017-05-04

**Authors:** Se Hee Lee, Min Young Jung, Jung-Hee Song, Moeun Lee, Ji Yoon Chang

**Affiliations:** Microbiology and Functionality Research Group, World Institute of Kimchi, Gwangju, Republic of Korea

## Abstract

*Lactobacillus curvatus* WiKim38 is a potential probiotic strain isolated from kimchi, a traditional Korean fermented food. The complete genome of the WiKim38 strain consisted of a circular chromosome of 1,940,170 bp in length with a G+C content of 41.93%.

## GENOME ANNOUNCEMENT

Lactic acid bacteria (LAB), which belong to the genus *Lactobacillus*, are found in fermented foods and have a positive influence on the maintenance of human health (1). The LAB strain *Lactobacillus curvatus* WiKim38 was isolated from the fermented cabbage food kimchi. This strain reportedly induces the production of the anti-inflammatory cytokine IL-10 in dendritic cells and alleviates dextran sulfate sodium–induced colitis in mice (2). In this study, we sequenced and fully assembled the genome of *L. curvatus* WiKim38 in order to elucidate the genetic elements about its probiotic potential.

Whole-genome sequencing of *L. curvatus* WiKim38 was performed using the Pacific Biosciences RS II sequencing platform (Pacific Biosciences, USA) with a 20-kb SMRTbell templates library (3) at ChunLab (Seoul, Republic of Korea), which generated 437,656,916 bp of data (73,573 reads; about 225.58-fold genome coverage). Sequences were assembled using the RS Hierarchical Genome Assembly Process (HGAP) protocol version 3.0, as available in SMRT Analysis software version 2.3.0. Functional categories were predicted using the Clusters of Orthologous Groups (COG) database of proteins on the BASys web server using GLIMMER gene prediction (4, 5). The complete genome of the WiKim38 strain consisted of a circular chromosome of 1,940,170 bp in length with a G+C content of 41.93%. This chromosome included 1,885 coding DNA sequences, 74 pseudogenes, 6 rRNA operons, and 65 tRNA genes. The identified genes were classified into functional categories based on their COG designation. Genome annotation revealed the presence of 190 genes involved in replication, recombination, and repair (those with COG abbreviation L were found to be the most abundant). The whole-genome sequence of *L. curvatus* WiKim38 was annotated using the NCBI Prokaryotic Genome Annotation Pipeline (PGAP) (6) and the Rapid Annotations using Subsystems Technology (RAST) server version 2.0 (7).

*Lactobacillus* spp. adhere to intestinal surfaces and participate in molecular cross-talk with the host via several molecular mechanisms (8–10). One of the most important criteria for the selection of potential probiotic species is the ability to adhere to and colonize the host gastrointestinal tract. This is expected to increase viability within the intestinal environment and thus maximize probiotic benefits (11, 12). The genomic analysis reveals that the genome contains the biosynthesis of d-alanyl-lipoteichoic acid encoded by *dltABCD* (LCW_RS08315 to RS08330) and EPS encoded by *epsHCDE* (LCW_RS02030, RS02390, RS02395, and RS02425). Several cell-surface proteins, such as sortase A (LCW_RS00355), and cell-wall-anchored proteins (LPxTG motif; LCW_RS08745 and RS09710) were also found in the genome. These results explain the adhesive ability of *L. curvatus* WiKim38 to intestinal epithelial cells and its survival strategy in the gut environment.

### Accession number(s).

The complete genome sequence of *L. curvatus* WiKim38 was deposited in GenBank under the accession number CP017124.
